# A qualitative meta-synthesis of facilitators and barriers to tuberculosis diagnosis and treatment in Nigeria

**DOI:** 10.1186/s12889-021-10173-5

**Published:** 2021-02-03

**Authors:** Charity Oga-Omenka, Lawrence Wakdet, Dick Menzies, Christina Zarowsky

**Affiliations:** 1 School of Public Health of the University of Montreal (ESPUM), Montreal, Canada; 2 Centre de Recherche en Santé Publique, Université de Montréal (CReSP), Montreal, Canada; 3 McGill University International TB Centre, Montreal, Quebec Canada; 4 Institute of Human Virology Nigeria, Kano, Nigeria; 5 Department of Epidemiology and Biostatistics, McGill University, Montreal, Canada; 6 School of Public Health, University of the Western Cape, Cape Town, South Africa

**Keywords:** TB case finding, TB treatment, Nigeria, Qualitative meta-synthesis, Barriers and facilitators

## Abstract

**Background:**

Despite progress in tuberculosis (TB) control globally, TB continues to be a leading cause of death from infectious diseases, claiming 1.2 million lives in 2018; 214,000 of these deaths were due to drug resistant strains. Of the estimated 10 million cases globally in 2018, 24% were in Africa, with Nigeria and South Africa making up most of these numbers.

Nigeria ranks 6th in the world for TB burden, with an estimated 4.3% multi-drug resistance in new cases. However, the country had one of the lowest case detection rates, estimated at 24% of incident cases in 2018 - well below the WHO STOP TB target of 84%. This rate highlights the need to understand contextual issues influencing tuberculosis management in Nigeria. Our synthesis was aimed at synthesizing qualitative evidence on factors influencing TB care in Nigeria.

**Methods:**

A three-stage thematic meta-synthesis of qualitative studies was used to identify barriers and facilitators to tuberculosis case finding and treatment in Nigeria. A search of eleven databases was conducted. The date of publication was limited to 2006 to June 2020. We analyzed articles using a three-stage process, resulting in coding, descriptive subthemes and analytical themes.

**Results:**

Our final synthesis of 10 articles resulted in several categories including community and family involvement, education and knowledge, attitudes and stigma, alternative care options, health system factors (including coverage and human resource), gender, and direct and indirect cost of care. These were grouped into three major themes: individual factors; interpersonal influences; and health system factors.

**Conclusion:**

Case finding and treatment for TB in Nigeria currently depends more on individual patients presenting voluntarily to the hospital for care, necessitating an understanding of patient behaviors towards TB diagnosis and treatment. Our synthesis has identified several related factors that shape patients’ behavior towards TB management at individual, community and health system levels that can inform future interventions.

## Background

Tuberculosis (TB) remains a global leading cause of death from a single infectious disease, infecting 10 million and killing an estimated 1.2 million people in 2018 [1]. It is caused by the bacillus *Mycobacterium tuberculosis*, and spread through minute droplets produced through coughing or sneezing [2, 3]. It typically affects the lungs (pulmonary TB) but can also affect other sites (extrapulmonary TB) [1]. People infected with the human immunodeficiency virus (HIV) are more prone to TB disease due to a weakened immune system [1, 4]. The risk of developing TB is 19 times higher for people living with HIV than in those HIV negative [1].

Some strains of TB have been resistant to first-line anti-TB medications, namely rifampicin or isoniazid. Drug resistant (DR-) TB therefore requires lengthy treatment with multiple, potentially toxic drugs that are up to five times costlier, and results in poorer treatment outcomes [1, 5–7]. Much of DR-TB results from human-made errors including poor management of drug-susceptible (DS-) TB infections, delayed, inadequate access or substandard TB medications, or poor adherence [8, 9]. DR-TB, estimated at 4.3% of new cases, was estimated to have claimed 214,000 lives in 2018 [1]. The mixture of high incidence rates of both TB and HIV infections in sub-Saharan Africa adds new levels of complications to diagnosis, emphasizing the need for coordinated and effective control strategies [1, 10].

TB case detection and access to medications are proven interventions in reducing the TB burdens of countries and are critical to meeting the global strategies to end TB. Several global initiatives have set targets towards ending the TB epidemic by 2030 [1, 11]. In September 2018, the United Nations held its first high-level meeting on TB with the goal of ending the TB epidemic by 2030, in line with the Sustainable Development Goal (SDG) Target 3.3 [11]. The WHO End TB Strategy 2016–2035 aims to reduce TB deaths by 90%, reduce new cases by 80% and ensure no family faces catastrophic costs due to TB [1]. The 90–90-90 Stop TB target aims to reach 90% of all people with TB, 90% of key populations while keeping a 90% treatment success rate [1].

In Nigeria, TB is a major public health problem. The WHO estimated the burden of TB in Nigeria in 2018, with 429,000 incident cases, to be the highest in Africa (and 6th highest globally) and resulting in 157,000 deaths [12]. However, the 2018 case notification and treatment rates for all forms of TB at only 24%, were among the lowest in the world [12]. Lack of access to healthcare and failure of healthcare workers to recognize symptoms and test for TB in patients are among some of the reasons for underdiagnosis of TB [1]. Additionally, a survey of TB patients in Nigeria revealed one of the highest catastrophic health costs (71%) among other high burden countries, mostly due to direct non-medical costs (transportation, lodging, and nutritional supplements) [1]. All these factors point to significant barriers in access to diagnosis and treatment in Nigeria, even though diagnosis and treatment for all forms of TB is provided for free [13].

The Nigerian TB Control Program has declared finding the missing TB cases as the most important priority for TB control for the upcoming years [14]. In order to meet any of these targets, there is an urgent need to understand contextual issues influencing TB management in Nigeria [1, 14, 15].

Several studies from Nigeria have identified barriers to TB care access [16–19], with only a few using qualitative design. The use of qualitative research is particularly necessary to thoroughly understand the contexts, complex relationships and patterns at play [20] at the different points in a patient’s pathway to TB care, as well as the perspectives and behaviors of patients and health providers and the complex relations in health care systems, and how these constitute barriers to TB diagnosis and treatment [21, 22]. A synthesis of qualitative research is useful in exploring questions about why interventions work or do not, and in what context; what the barriers and facilitators are to accessing health care and the impact of these on people, their behaviors and experiences [23]. In order to understand the contextual factors that influence access to care, there is a need to bring together, summarize and explore new meanings from the available qualitative data [24–26].

Our systematic review of qualitative studies explores the barriers and facilitators of TB healthcare in Nigeria using a meta-synthesis approach [27]. The research question guiding this meta-synthesis is: What does qualitative research tell us about the barriers and facilitators to TB care in Nigeria?

## Methods

### Overview

Our thematic synthesis explored barriers and facilitators to TB programs at the individual, community and at the health system levels [28, 29]. The steps in a meta-synthesis involve identifying the research question and relevant studies, appraising the studies for quality and synthesizing the studies [30]. After the process of literature selection and quality assessment, this study applied a thematic approach to qualitative meta-synthesis, as described by Thomas and Harden [27]. The thematic synthesis is applied in three steps beginning with the creation of codes from the original study findings, further organization into descriptive subthemes and finally development of analytical themes [27, 31].

### Step 1: identifying the research question

Our research question aligns with the operational and public health research priorities of the WHO International Roadmap for TB Research and the Nigerian TB Program to target barriers in the scale up of TB diagnosis, treatment and care [14, 32, 33]. The WHO identified these priorities through review of evidence, expert meetings and consultations with stakeholders and working groups of the Stop TB partnership [32].

The research question was framed using the **SPIDER** approach as recommended by Cooke et al. [34]:

- **Sample**: Tuberculosis patients in Nigeria
- **Phenomenon of Interest**: Access to diagnosis and treatment of TB
- **Design**: Meta-synthesis of research using interviews, focus group discussions, observation, in-depth or key informant interviews
- **Evaluation**: The reported barriers and facilitators
- **Research type**: Qualitative research

Our SPIDER-generated research question: *What does qualitative research tell us about the barriers and facilitators to diagnosis and treatment access for TB patients in Nigeria?*

### Step 2: data sources and identification

We created a search strategy by identifying four key themes in the research question: tuberculosis, access, qualitative and Nigeria. Search terms and variations were generated based on the themes. These can be seen in the Table 1: Medline Search Strategy.

**Table 1:** Medline Search Strategy

1	Tuberculosis, Pulmonary/ Or *Mycobacterium tuberculosis*/ Or Tuberculosis/ Or Latent Tuberculosis/ Or Tuberculosis, Multidrug-Resistant/ Or Extensively Drug-Resistant Tuberculosis/
2	limit 1 to yr = “2016 -Current”
3	(delay or barrier or diagnostic error* or access or late).mp. [mp = title, abstract, original title, name of substance word, subject heading word, keyword heading word, protocol supplementary concept word, rare disease supplementary concept word, unique identifier, synonyms]
4	limit 3 to yr = “2016 -Current”
5	(qualitative or interview$ or focus group$ or phenomenology or ethnograph$ or grounded theory or observation$ or field stud$ or case stud$).mp. [mp = title, abstract, original title, name of substance word, subject heading word, keyword heading word, protocol supplementary concept word, rare disease supplementary concept word, unique identifier, synonyms]
6	limit 5 to yr = “2016 -Current”
7	NIGERIA/
8	limit 7 to yr = “2016 -Current”
9	2 and 4 and 6 and 8

Eleven databases searched were searched- Medline, Scopus, EBM Review, Web of Science, Pubmed, Embase, CINAHL, Global Health, African Journal Online (AJOL), the International Journal for TB and Lung Disease (IJLTD) and Google Scholar. A reference list search for included studies did not yield any additional studies. Search results were exported to EndNote®. The date of publication was limited from January 2006 (2006 being the first publication year for the WHO *Guidelines for the programmatic management of drug-resistant tuberculosis*) to the date of search, which was 18th of August 2018. The search was rerun on the 30th of June 2020. There were no language restrictions.

## Step 3: study selection and quality assessment

**Fig. 1:**
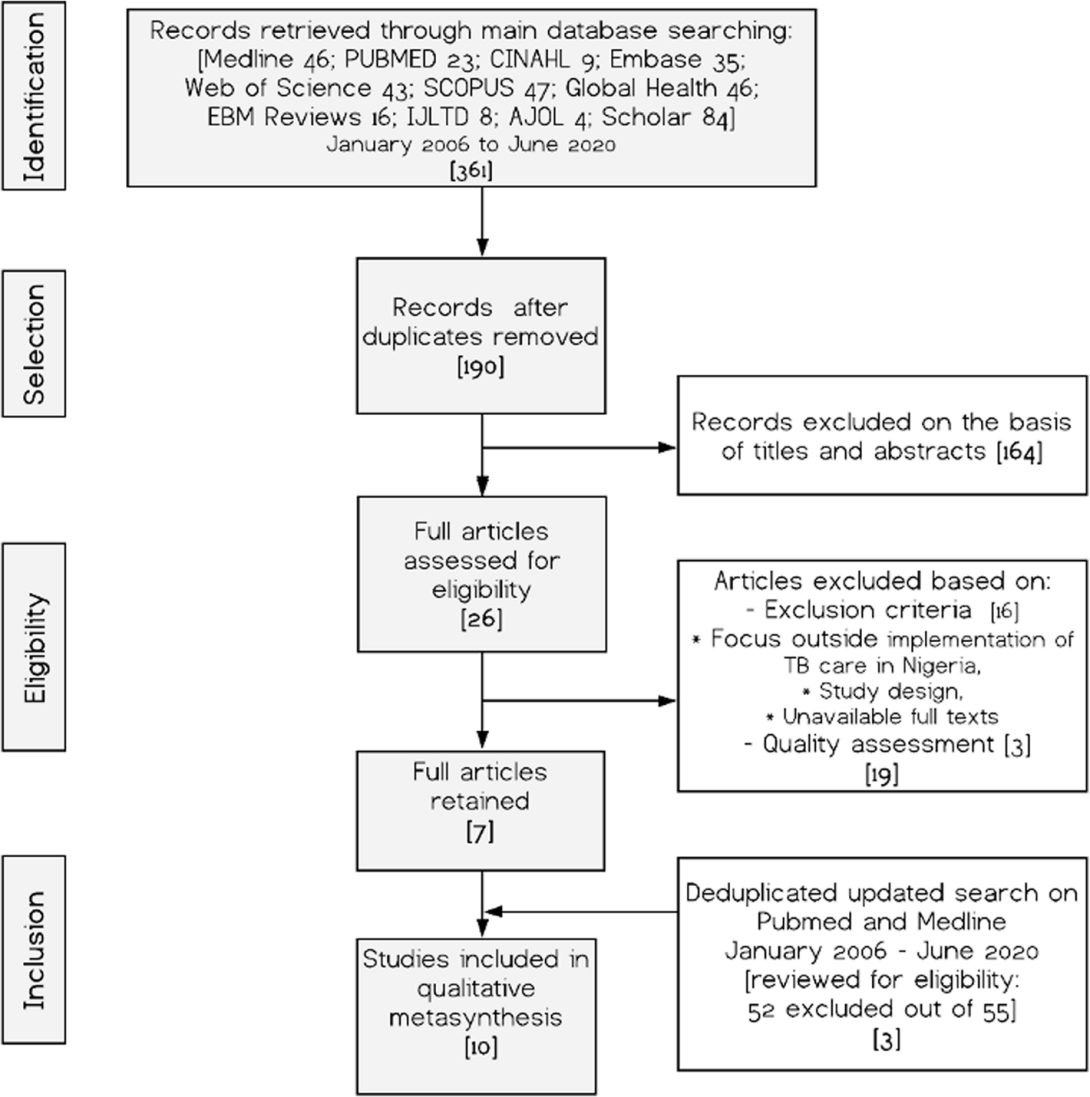
PRISMA Diagram of Selection Process

The citations imported into Endnote® were screened in a stepwise approach starting with removal of duplicates, followed by the review of titles, abstracts, and finally the full text based on the inclusion and exclusion criteria. This process is shown in Fig. 1.

### Inclusion criteria

- Studies focusing on case finding and treatment of TB in Nigeria
- Studies published from 2006 to June 2020
- All languages

### Exclusion criteria

- Abstracts without full texts
- Focus outside Nigeria
- Not peer reviewed
- Absence of qualitative data

A quality assessment was performed independently by two authors (COO and LW), using the simplified criteria for qualitative research, recommended by Murphy et al. [35]. This involved two initial criteria and seven screening questions to determine the credibility and relevance of the studies [35, 36]. Results of the quality assessments were discussed, and differences resolved by consensus.

### Step 4: thematic synthesis

As described by Thomas and Harden, the process of a thematic synthesis has three stages – free line-by-line coding of the text within the ‘Results’ sections of the primary studies, creation of descriptive subthemes by organizing the codes into related areas, and finally, the generation of analytical themes across the set of retained studies [27]. The descriptive subthemes are similar to that found in the primary studies, but the analytical themes represent the new interpretation generated from those [27, 31, 37]. All texts of results were read, line after line, and the content of each text fragment interpreted. All sentences were then copied into the Quirkos database, except for one, [38], which examined community initiatives for health interventions and not just for TB. For this, only sections within the results that were relevant for TB disease were selected. The next step was a sentence-by-sentence coding base on meaning and content, with some sentences getting more than one code. This process resulted in a total of 21 initial codes. The process of developing the descriptive subthemes involved looking at the similarities and differences between the codes and grouping them into a hierarchical tree structure. New terms were used to describe the meanings and contexts of groups of initial codes. This process resulted in a hierarchical structure with a total of 11 descriptive subthemes. Three analytical or ‘higher order’ [39] themes were created by interpreting the relationships between themes across studies and in relation to the research question. This was done using the Quirkos software.

## Results

### Study characteristics

There were 10 studies from Nigeria that explored factors that impact access to TB care from a qualitative perspective, 9 qualitative studies and 1 mixed method. The characteristics of the 10 studies synthesized are presented in Table 2.

**Table 2:** Overview of selected studies

	First Author, Year	Title	Data collection	Method of data analysis	Main themes
1	Adejumo, 2020 [40]	Challenges of Tuberculosis Control in Lagos State, Nigeria: A Qualitative Study of Health-Care Providers’ Perspectives	34 in-depth interviews with health workers	Data coded deductively into previously dentified themes	Challenges with TB management and supervision, laboratory tests, DOTS providers’ training, and work overload
2	Ajayi, 2013 [38]	Assessing resources for implementing a community directed intervention (CDI) strategy in delivering multiple health interventions in urban poor communities in Southwestern Nigeria: a qualitative study	12 Focus group discussions and 73 key informant interviews (KIIs) with stakeholders	Content analysis- inductive and deductive	Community resources can facilitate access to health care
3	Bieh, 2017 [41]	Hospitalized care for MDR-TB in Port Harcourt, Nigeria: a qualitative study	2 gender based FGDs and 11 in-depth interviews with patients	Transcription of data, coding and thematic assembly and analysis	Patient-centered care improves access and removes stigma
4	Ogbuabor, 2020 [42]	Through service providers’ eyes: health systems factors affecting implementation of tuberculosis control in Enugu State, South Eastern Nigeria	23 in-depth interviews with health workers	Framework approach	Leadership and governance, health financing and human resources, supply chain system (technology), health information system and service delivery
5	Olukolade, 2017 [43]	Role of treatment supporters beyond monitoring daily drug intake for TB-patients: Findings from a qualitative study in Nigeria	2 FGDs, 15 KIIs and IDIs	Data transcription and content analysis	Patient nominated treatment supporter and patient centered approach to TB Therapy very crucial
6	Okeibunor, 2006 [44]	Barriers to care seeking in directly observed therapy short-course (DOTS) clinics and tuberculosis control in southern Nigeria: a qualitative analysis	24 in-depth interviews & 24 FGDs	Themes were developed in the form of codes and further summarized ethno-graphically	Perceived causes of TB infection, perceived high costs & quality of care prevent patients from accessing available services
7	Onyeneho, 2010 [45]	Is there a role for patent medicine vendors (PMVs) in tuberculosis control in southern Nigeria?	17 interviews each with PMV and community leaders	Developing, describing and interpreting codes	Knowledge and practice about TB, referral practices, awareness of TB clinics, involvement in detection of TB cases and attitudes towards involvement of PMVs in TB control
8	Oshi, 2016 [46]	Gender-related factors influencing women’s health seeking for tuberculosis care in Ebonyi state, Nigeria	56 interviews – with 46 women and 10 men from 6 communities	Cross-case analysis of key themes	Socio-cultural & economic factors weaken women’s access to health care
9	Ushie, 2012 [47]	The paradox of family support: Concerns of tuberculosis-infected HIV patients about involving family and friends in their treatment.	8 FGD, 21 In-depth Interviews, 4Case histories	Thematic analysis	Family support promotes adherence
10	Ukwaja et al. (2017) [48]	Sustaining the DOTS’: stakeholders’ experience of a social protection intervention for TB in Nigeria**.**	103 key Informant interview, 2 FGD, 10 In-depth interviews	Thematic content analysis until data saturation	Patients and health workers recorded positive outcomes with financial inducements

A variety of methods were used in data collection including in-depth interviews, focus group discussions, semi-structured interviews and key informant interviews. Sample size ranged from 24 to 221.

The ten studies differed in focus even though all directly or indirectly explored factors influencing diagnosis and treatment to care- including barriers to direct observable treatment short course (DOTS), the WHO endorsed system of TB care [40, 43, 44, 48]; the role of family members [41, 44, 46, 47], patent medicine vendors (PMVs) [45] and treatment supporters [43]; particular focus on gender-based factors [46], care pathways [44], challenges of drug-resistant TB care [41] and TB/HIV co-infection [47]; as well as community-level interventions with some particular reference to TB [38].

### Quality assessment

The quality assessment for the selected studies are presented in Table 3. Three out of ten studies were classified as “A”, and the remaining 8 classified as “B”, using the Murphy criteria [35].

**Table 3:** Quality Assessment

Study	Credibility					Relevance		Score
	Data collection	Auditability	Reflexivity	Negative cases	Fair dealing	Transferability	Analytic generalization	
Adejumo 2020 [40]	x	x				x	x	B
Ajayi 2013 [38]	x	x	x		x	x	x	A
Bieh et al., 2017 [41]	x	x	x		x	x	x	A
Ogbuabor 2020 [42]	x	x			x	x	x	B
Olukolade et al., 2017 [43]	x	x			x	x		B
Okeibunor et al., 2006 [44]	x	x			x	x	x	B
Onyeneho 2010 [45]	x	x			x	x	x	B
Oshi et al., 2016 [46]	x	x			x	x	x	B
Ushie & Jegede 2012 [47]	x	x			x	x	x	B
*Ukwaja* et al *2017* [48]	x	x	x		x	x	x	A
*1-2 points = C, 3-5 points = B and 6-7 points = A*								
**Quality assessment questions**								
***Credibility***								
Data collection	• Were explanations of sampling strategies and data collection methods provided?							
Auditability	• Was the method of data analysis described and enough data displayed to allow the reader to determine whether the interpretations made by the researcher is supported by the data?							
Reflexivity	• Did the authors acknowledge the influence of the research process and the presence of the researcher including the role of prior biases, assumptions and experience, on the collected data?							
Negative cases	• Has appropriate attention been given to contradictory data? Are negative cases taken into account?							
Fair dealing	• Did the authors explore alternative, plausible explanations for the data collected and incorporate a range of different perspectives?							
***Relevance***								
Transferability	• Did the authors provide information regarding participants, setting and context so that the reader might be able to determine the relevance of the findings to other settings (transferability)?							
Analytic generalization	• Did the authors discuss findings within a broader context, propose generalization of findings and/or suggest a direction for future research?							

### Description of themes

**Fig. 2:**
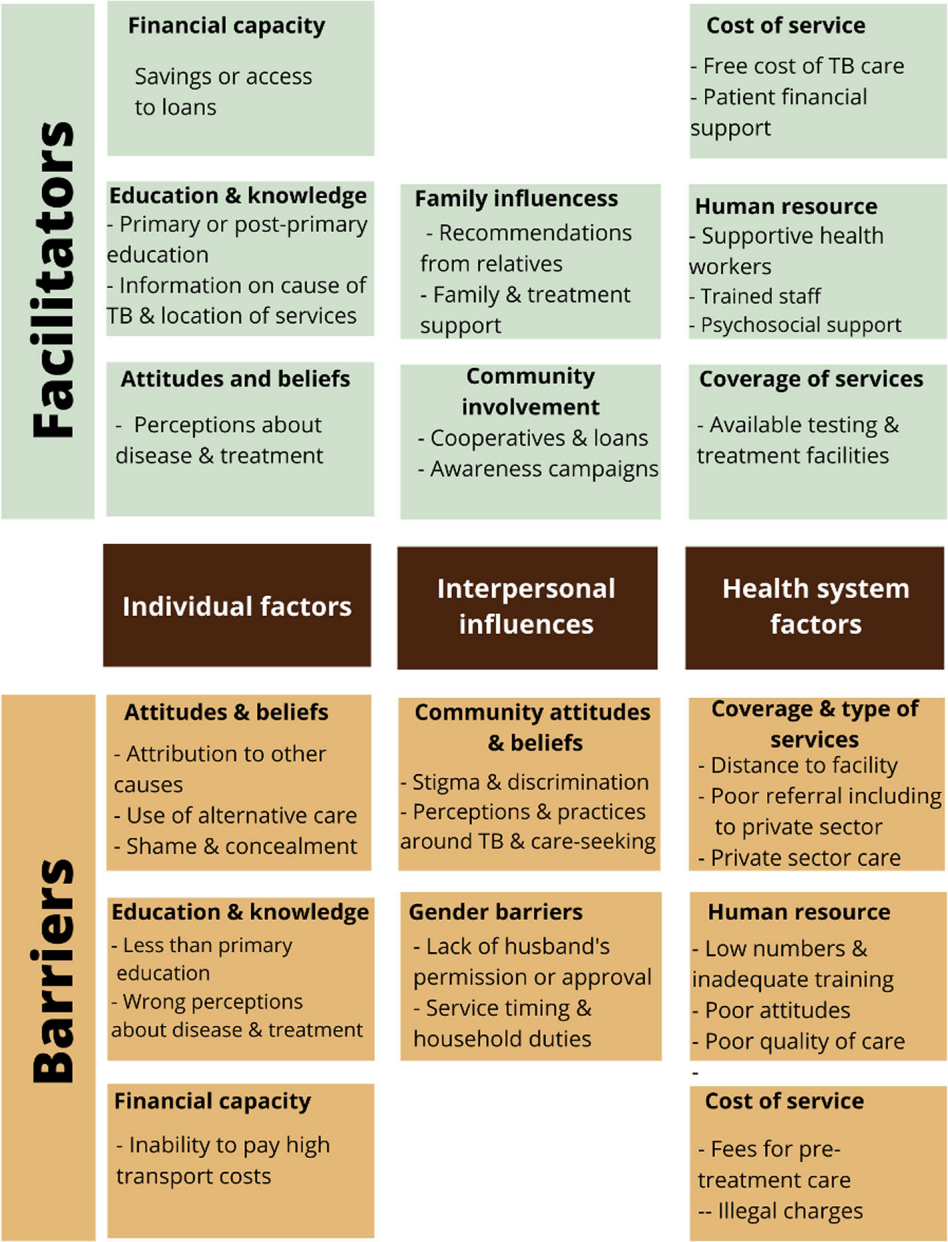
Diagram showing barriers and facilitators associated with each theme and subthemes

Our synthesis yielded three main themes and eleven inter-related sub-themes *(*Fig. 2*).* On the individual level, financial capacity, education and knowledge, as well as attitudes and beliefs were facilitators or barriers. On the interpersonal level, family influences and community involvement were facilitators, while negative community attitudes and beliefs, especially towards public sector TB care, and harmful gender norms, were barriers. At the facility level, cost of service, human resource and coverage and type of services were either facilitators or barriers. We also discuss the stage of care - diagnosis or treatment – the access factor was more prevalent.

Each of the three themes are presented in more detail below.

### Individual factors

This theme reflects the individual-level factors that determine the use of healthcare. It emanated from three descriptive subthemes of financial capacity, education and knowledge, attitudes and beliefs.

Cost of treatment, particularly indirect costs such as relating to transport or additional fees, were cited by participants as a major barrier to both diagnosis and treatment. For example, one participant in the Oshi et al., 2016 study stated that *“There are very few women in my community who can afford the costs of transportation to the hospital and to pay the hospital fees. Maybe the traders! Even then, if they are not cautious taking money from their business to go for treatment will spoil [ruin] their business...”* (Interview with 29 years old female with secondary education) [46]. In a different study [38], a participant discussed mentioned how *“Many women go to faith healers now not because they do not know that it is better to have treatment in the hospital, but because they cannot afford it. Many of our women are not employed …*” [38].

However, patients who received family support, as discussed below, were better able to mitigate the cost barrier.

Several codes were related to the patient’s education and knowledge. Participants noted that attitudes and beliefs hindered correct diagnosis. Knowledge about the cause of TB or the availability of free care, negative perceptions of public sector care or a preference for alternative care in the private sector were recurring barriers to diagnosis.

*“It is only a prophet who can destroy the powers of the witch. So, such persons who have TB can only be cured through prayers by powerful prophets”* (41 years old female, uneducated farmer) [46].

*‘It took some time before I went to get drugs for this illness... I went to* two prayer houses yet I did not get better ...so I started taking herbal treatment’ (FGD, 42-year-old male) [48].

For some patients, the use of alternative treatments, or the reason for delayed care-seeking were due to financial constraints. This was a particular barrier to diagnosis.

“*For the first two months when I started coughing, it was very hard for me because I had no money to go to the chemist... even after I visited the chemist and got some drugs the cough persisted*” (FGD, 65-year-old male) [48].

“*I did not have any money to buy drugs when my illness started...so I started taking herbal remedies*” (FGD, 58-year- old female) [48].

Some of the themes were inter-related, for example uneducated patients were more likely to delay treatment in favor of prayer houses and traditional healers.

*“Health seeking depends on the level of education. The [uneducated] would delay going to hospital. They first use local herbs.” “Patients first move to chemists and herbalists and finally hospital if they cannot get cure from these sources. Where they go first depends on whether they believe TB is man-made, natural, or caused by witchcraft.”* [44]*.*

A few patients complained about the fact that most available health resources in their immediate localities were focused on pregnant women and children, alienating males and other women. This perception of public healthcare could have been a barrier to diagnosis, especially for males.

*“All the health programmes in this community are designed for women and children. Is there nothing that can be done for adult males and females? If we are sick and not treated, we will transfer the sickness to the children* [38].

### Interpersonal influences

This theme refers to the effect of the family and community on the decision-making processes of people in need of care, ranging from codes on stigma and discrimination to community awareness campaigns. It is also reflected in the extent of deference women were expected to have towards their husbands when it came to health seeking decisions.

Factors like stigma and discrimination affected women differently from men, as some participants were quoted saying a female TB patient would be a pariah and would be unlikely to get suitors or could possibly be divorced as a result of her illness. Also, a husband who attribute TB to other causes or who have negative perceptions about its management could potentially prevent his wife from accessing care. These gender norms were mentioned by different participants. One participant said, *“Women should be submissive to their husbands. A woman who knows more than her husband portends danger to the community. If a man says the medicine is good, then it is good. If he says the medicine is not good, who is the wife to disagree*.” (elderly, female, farmer) [46]. Another mentioned that, “*… The ability of a woman to pay for hospital treatment does not mean that she can just get up and go to the hospital without her husband’s permission.”* [46]*.* In another study, a participant mentioned that marriages were breaking up because of one partner’s infection. *“The community leader … argued that traditionally, infection with TB is not grounds for divorce, but spouses often cloak the real reason for seeking separation.*. *.”* [44]*.*

On the other hand, family and community support were facilitators to initiating and adhering to treatment. This support was sometimes financial and this enabled patients to continue accessing treatment.

*“For me it was very traumatic to start taking drugs I have not been used to... but I got so many encouragements, people around me, .... And probably to be frank, if not for their own encouragement and everything, I would have stopped; because this is my last month, I wouldn’t have taken it this far, I would have stopped, but they encouraged me that I had to complete the period of medication”* Female TB Patient, Asokoro [43].

“*My son and wife have been very helpful...they accompany and support me to go to the clinic and to take my drugs”* (IDI, 50-year-old male) [48].

“*Whenever I am seating lonely and quiet, my husband usually comes to me and asks: ‘why am I seating quiet, am I alright? And whenever my drugs are finished, he goes and gets them for me and he advises me to take the drugs*”, Female TB Patient Interview, [43].

*“My difficulty is getting transportation to get to this place (DOTS centre). I will go and meet my brother to tell him that I am supposed to go and take drugs then he will find me some money for transportation*”, Male TB Patient [43].

For some, having a knowledgeable person in the community, sometimes healthcare workers, direct them to the right public facility for TB care were facilitators of diagnosis.

“*I visited all the chemists in my community and took several medication, yet the cough kept increasing...I visited the health centre twice before the nurse said I may have TB I should come to this hospital*” (IDI, 35-year-old female) [48].

### Health system factors

Health system barriers were a recurring theme in all the studies. They emerged from codes around poor coverage of services, weak referral system, low numbers and poor skills in health workers, poor attitude of staff and quality of care, corrupt staff demanding illegal charges from patients and the relatively high cost of patient cards. These codes were grouped into three descriptive subthemes- coverage and type of services, human resource, and cost of service.

Coverage and opening hours were cited as barriers, mostly to diagnosis but sometimes in relation to treatment access.

*“If they [hospitals] want to help us they should make their opening hours flexible so that we [women] can also go there in the evening hours, after the days’ work. That will be more convenient for women.”* [46].

Poor coverage was also linked to high transportation costs and the reason why patients were choosing alternative care in the private sector. These were mostly barriers to diagnosis.

*“There is no general hospital around. The nearest one … is far from here. Many people don’t want to go there because of cost of the transportation and services. People treat themselves at home or go to herbalists. Only very few people go to private hospitals as they are very expensive* [38].

The attitude of healthcare workers created barriers to diagnosis for a number of patients. This was a recurring theme across several studies. One participant said, *“Even some nurses and medical workers treated us like we are not fit to live again. They keep a distance when they want to communicate with us. If you come closer, they will shout go! go!! go!!! ........ The feeling of stigma is very difficult. I felt like the worst person on earth having MDR-TB” (IDI, 29 years old male* [41]. A healthcare worker in another study confirmed this discriminatory treatment of patients, *“…. I have been sending my patients for a laboratory test at a particular hospital, but she refused to go because of the way she was treated the last time she went. I decided to visit the place myself to see with my eyes what was going on. You cannot imagine what I saw. Immediately they (the health workers) saw me approaching them, they shouted at me to go back as I walked in not knowing I am a DOTS Provider. I can imagine what the patients go through …*.” *(DOTS Provider)* [40].

Healthcare workers, on their part, complained of shortage of workers, unbearable workloads, inadequate training and a lack of laboratory resources. These challenges were mostly barriers to diagnosis, and sometimes to treatment as well.

“… *For me as an example, I am the clinician in my facility. I am expected to go on outreach [es], consultation of patients that come to my facility is my responsibility. I am the DOTS provider. I used to have 22, now I have 27 centers under me to supervise. Then how do I share myself*, …” (TB Supervisor) [40].

“*My workload is high. It is not only TB services that I provide. I am also the anti-retroviral therapy focal person*” [42].

Patent medicine vendors are readily available in the communities and see a lot of clients with coughs. However, many PMVs were not trained on TB control nor were aware of TB DOTS centers but were overwhelmingly eager to be part of the TB control effort [45]. The use of alternative care in the private sector was always a barrier, due to the poor linkage between the public and private sector. Several reasons contributed to the use of the PMVs and some of these linked back to individual factors. They included poor coverage of health services, patients in geographical locations with poor accessibility where only PMVs were available, perceived high costs of care in the public sector, even though TB services were supposedly free.

*“Patent medicine vendors are the main source of healthcare service delivery in this community. Many people go to PMVs because medicines are not readily available at the health centres and if available, they are costlier because patients have to pay for other services such as a consultation when they go to the hospital”* [38]*.*

*“We are in the local communities, a very interior part of the community, and we deal with people of the local communities; hence, such people come to the patent medicine shop to re- quest, “sir, do you have something like this”* (PMV practitioner) [45].

Some of the PMVs were not aware of how to diagnose TB or the availability of free TB services in the public healthcare system.

“… *I have not heard about it (DOTS clinic). I am not aware of DOTS clinic but if it is evolved, it will help in the control of people with tuberculosis in our environment*” Interview with an official of a local PMV association [45].

*“They (PMVs) should be informed, involved, educated, and trained.... You will know what you have learnt, and if anything comes up, you will tell the person”* (PMV leader) [45].

Other factors that were mentioned include absence of doctors at community levels and illegal charges demanded from patients. Several patients alleged that healthcare workers were diverting free medications from the TB centres to their private health centres, where they now charge the patients. As this was mainly from one article, we were not able to ascertain if this problem was widespread.

“*Attitude of health workers, cost and distance are the issues here. Health workers at the DOTS clinics can hardly be seen, sometimes for a month or more. The health workers have their private Chemists/consulting shops where they treat their private patients. They sometimes divert clinic resources for that purpose*” (FGD with women) [44].

“*Health workers at DOTS clinic, all have consulting shops outside the hospital. Drugs are not free. Attitude of health workers toward patients is influenced by amount of money the patient has*” (FGD with men) [44].

Key facilitators included the financial support provided to the patient by the program.

*“I thank you for providing us this money...most times when I want to come to the hospital I borrow money for transportation then when I collect the money I will go and pay back”* (IDI, 45-year-old female) [48].

## Discussion

This meta-synthesis looked at barriers and facilitators to TB diagnosis and treatment in Nigeria and resulted in three major themes, centered on individual, interpersonal influences, and health systems factors. These themes were common across different studies, irrespective of type of study and data collection method. They were also inter-related even though presented here separately. The findings of this synthesis are in line with several other studies, within and outside Nigeria.

For example, studies on TB control in Nigeria show operational challenges occurring at patient and health system levels. Reported barriers include beliefs about causes of TB, knowledge of treatment duration and benefits, socioeconomic status, literacy, stigma, hidden treatment costs, distance from the clinic, access to health care, and health worker attitude and knowledge [17, 49].

Specifically, other studies and reviews, including from elsewhere in Africa, identify *patient level* barriers as cost of care, fear of stigma, distance from facility, worsening disease, inadequate knowledge about the disease, and perceptions of poor quality of care at hospitals [50–55]. Facilitators to patient TB care-seeking behavior have been identified in literature as knowledge of TB and HIV disease and treatment [54, 56].

At the *community level*, other studies also identified awareness and screening campaigns as facilitators [56–58]. These agree with findings of studies from several African studies in Zimbabwe, Malawi and Ethiopia, where active case finding in the community, e.g. using mobile vans to be very effective even in communities in close proximity to a hospital [59–64].

At the *health system level*, barriers like poor tracking of patients, delays in access to testing, staff shortages and work overload/overtime, inadequate health worker knowledge of transmission, misdiagnosis, poor infection control, staff shortages, overwhelming workloads, additional testing required and lengthy triage procedures have been identified [51–53, 56, 57, 65–68].

On the other hand, facilitators in health systems are patient financial support, quick testing time, appropriate counseling and testing, patient tracking, health worker training, quick and efficient workflows, sufficient staffing, free and confidential TB services [50, 51, 54, 56, 57, 69–71].

Authors of selected articles made several recommendations for tackling identified barriers. These included training, supervision and logistical support for healthcare workers, as well as resource mobilisation and hiring of new health workers [40, 42, 44]; training and engagement of patent medicine dealers by the National TB program [45]; financial, family and community support, as well as home-based care wherever possible for patients [38, 41, 43, 47, 48]; women empowering policies, programmes and interventions targeting harmful gender norms with the aim of increasing women’s access to TB services [46].

### Meta-synthesis limitation

The main limitation of our study is that we chose to include most of the studies that met our inclusion criteria, sometimes in spite of quality, and low methodological rigor. This was done to include as much available data as possible, due to a low availability of qualitative research on barriers to TB care from Nigeria. As a result of this, the identified themes are subject to the limitations, rigor and quality of the original articles. This may limit the transferability of our findings. However, we ensured that all selected studies had relevant data on barriers to TB care in Nigeria. Another limitation is the sparsity of data relating to several aspects of TB care identified in literature as being more difficult, including pediatric or drug-resistant TB diagnosis and treatment.

## Conclusions

This synthesis highlights a number of factors influencing access to diagnosis and treatment of TB in Nigeria, including attitudes and beliefs, financial capacity, education and knowledge on the individual level; community attitudes and beliefs, family influences, including negative gender norms, community involvement and private sector care on the community level; and coverage, human resource and cost of service on the health system level.

Based on these findings, interventions are needed to improve case finding, for example, increasing patient education and community awareness to modify harmful perceptions on TB disease and management. This could potentially improve diagnosis and treatment rates by reducing the time and expenses spent by patients seeking care outside facilities equipped to diagnose and treat TB cases in Nigeria. Also, measures are needed to improve health worker attitudes and the quality of care they provide.

## Data Availability

All data analysed for this study are included or referenced in this published article [and its supplementary information files].

## Abbreviations

AJOL: African journal online
TB: Tuberculosis
CINAHL: Cumulative index of nursing and allied health literature
DR-TB: Drug resistant tuberculosis
EBM: Evidence-based medicine reviews
FGD: Focus group discussion
HIV: Human immunodeficiency virus
IJLTD: International journal for TB and lung disease
PMV: Patent medicine vendors
PRISMA: Preferred reporting items for systematic reviews and meta-analyses
SPIDER: Sample, phenomenon of interest, design, evaluation, research type
WHO: World health Organization
